# Robotic transanal excision of rectal lesions: expert perspective and literature review

**DOI:** 10.1007/s11701-022-01469-8

**Published:** 2022-10-16

**Authors:** Sarah Watanaskul, Marisa E. Schwab, Hueylan Chern, Madhulika Varma, Ankit Sarin

**Affiliations:** 1 School of Medicine, University of California San Francisco, San Francisco, CA USA; 2 Department of Surgery, University of California San Francisco, 550 16th Street, San Francisco, CA 94143 USA

**Keywords:** Robotic, Transanal resection, Rectal lesion, TAMIS

## Abstract

Transanal excision of benign lesions, moderately or well-differentiated rectal T1 adenocarcinomas is typically completed via transanal endoscopic microsurgery (TEM) or laparoscopic transanal minimally invasive surgery (TAMIS). Robotic platforms provide ergonomic comfort in an enclosed space, enhanced range of motion, and superior 3D visualization. This study sought to perform a literature review of robotic TAMIS (R-TAMIS) and provide expert commentary on the technique. A Pubmed literature search was performed. Study design, robot type, indication, techniques compared, surgical margins, conversion, complications, operative time, estimated blood loss, patient positioning, and defect closure were collected from included articles. Expert opinion on pre-operative planning, technical details, and possible pitfalls was provided, with an accompanying video. Twelve articles published between 2013 and 2022 were included. Five were case reports, three case series, two prospective cohort studies, one retrospective cohort study, and one Phase II trial. The Da Vinci Si (*n* = 3), Xi (*n* = 2), single port (*n* = 3) and flex robotic system (*n* = 2) were used. Five studies reported negative surgical margins, one reported positive margins, and six did not comment. Operating room time ranged from 45 to 552 min and EBL ranged from 0 to 100 mL. Patient positioning varied based on lesion location but included supine, prone, modified lithotomy, and prone jackknife positions. 11/12 studies reported defect closure, most commonly with V-Loc absorbable suture. We recommend pre-operative MRI abdomen/pelvis, digital rectal exam, and rigid proctoscopy; prone jackknife patient positioning to avoid collisions with robotic arms; and defect closure of full-thickness excisions with backhanded running V-Loc suture.

## Introduction

Robotic surgery has become an increasingly popular tool for colorectal surgeons in the United States. A population-based study of Medicare beneficiaries from 2010 to 2016 showed an overall shift toward greater proportional use of robotic elective colectomy from 0.7% in 2010 to 10.9% in 2016 [1]. While the robotic approach has become common for surgeries such as colectomies and proctectomies, it remains a novel technique for transanal excision of rectal lesions. Rectal lesions amenable to transanal excision include benign lesions, small neuroendocrine tumors of the rectum, and rectal T1 adenocarcinomas that are moderately and well differentiated, (i.e. without lymphovascular or perineural invasion) [2].

These lesions have been traditionally excised by open technique if close to the anal verge or via transanal endoscopic microsurgery (TEM) or laparoscopic transanal minimally invasive surgery (TAMIS) if lesions are more proximal, but these methods are characterized by limited maneuverability and visualization. Robotic platforms offer several advantages for this surgery, including the ability to work in a small space with improved ergonomics and enhanced range of motion as well as superior 3D visualization [3, 4]. In a prior study [5], we compared robotic with laparoscopic TAMIS and traditional TEM, finding that all ten patients who underwent R-TAMIS procedures had negative surgical margins, as opposed to 10/13 in the TEM group and 5/6 in the TAMIS group. Furthermore, median operative times were lower for R-TAMIS (76 min) compared to TEM (110 min) and TAMIS (105 min).

In this study, we sought to review the literature on robotic TAMIS (R-TAMIS) for rectal lesions and provide practical tips and tricks for surgeons interested in adding this technique to their clinical repertoire.

## Materials and methods

### Literature review

An English language literature review was conducted in the PubMed database using combinations of the following search terms: “robot”, “transanal”, “rectal”, “excision”, “resection.” All robotic platforms were included in the search. This yielded 43 articles. After reading the abstracts, 13 articles of relevance were included. Two authors (SW, MS) independently reviewed the full manuscripts, and 11 articles were included. Studies pertaining to a different operation (such as abdominoperineal resection), review articles, and studies without human data were excluded. The references of the included articles were then reviewed to ensure no additional articles not captured in the PubMed search had been missed. Ultimately, 12 articles were included.

The following variables were available and reviewed from the included studies: study design, robot type, indication for resection, other techniques compared, surgical margins, conversion to other technique, complications, operative time, estimated blood loss (EBL), patient positioning, and whether the surgical defect was closed.

### Expert perspective

A colorectal trained surgeon (AS) with 8 years of expertise in robotic surgery recorded a robotic transanal operation that illustrates the principles of this surgical technique (see linked video). A 63-year-old woman experiencing hematochezia was found to have a large rectal lesion on colonoscopy. This was diagnosed as a villous adenoma with low-grade dysplasia on biopsy. Pre-operative rigid proctoscopy located the lesion to the posterior wall at 8 cm from the anal verge, and she underwent a R-TAMIS procedure as shown. The final pathology was villous adenoma with high-grade dysplasia with negative surgical margins. She was discharged on post-operative day 1 and was doing well with no functional impairments at 1-month follow-up.

Expert opinion regarding pre-operative planning, intra-operative positioning and technical details, and possible pitfalls was provided.

The study was approved by the University of California San Francisco’s Institutional Review Board (IRB) Study Number 18-26,677.

## Results

### Literature review

Twelve articles published between 2013 and 2022 were included (Table 1). Of the included articles, five were case reports, three were case series, two were prospective cohort studies, one was a retrospective cohort study, and one was a Phase II clinical trial. Robotic platforms included the Da Vinci Si (*n* = 3), Da Vinci Xi (*n* = 2), Da Vinci single port (*n* = 3), and the flex robotic system (*n* = 2). One article compared patient outcomes from procedures with the Da Vinci Si versus flex robotic system, and another article did not specify which version of the Da Vinci system they used. The study sample size ranged from 1 to 26 patients, with a total of 114 pooled patients.

**Table 1:** Literature review

Author year	*n*	Study design	Robot	Indications	Conversion to other technique	Complications	OR time	EBL	Positioning	Defect closed
Morino 2022 [6]	26	Retrospective cohort	Flex robotic system	Benign adenoma, early rectal adenocarcinoma	TEO (*n* = 6)	Bleeding requiring transfusion (*n* = 2), local recurrence (*n* = 2)	Median: 115 min (range: 45–360)	Negligible	Supine (*n* = 16), Prone (*n* = 10)	Yes
Marks 2021 [7]	26	Phase II clinical trial	DV single port	Benign polyp, select rectal cancer (T1, < / = T2 after neoadjuvant therapy)	TEM (*n* = 2), laparoscopic low anterior resection (*n* = 1)	Pelvic abscess (*n* = 1), wound dehiscence (*n* = 3), peritoneal entry (*n* = 5)	Avg: 198.8 min (87–552)	Avg: 24.2 mL (range 0-185 mL)	Supine modified lithotomy (*n* = 25), prone (*n* = 1)	Yes
Studniarek 2021 [13]	1	Case report	DV single port	Rectal neuroendocrine tumor	No	–	–	–	Right lateral decubitus	–
Lo 2022 [11]	16	Case series	DV Xi	Tubulovillous adenoma, rectal adenocarcinoma	No	Incontinence (*n* = 1), presacral abscess (*n* = 1), rectal abscess (*n* = 1)	Avg: 87 min (IQR 54.75- 109.5)	Avg 17.5 mL (IQR 8.75-20 mL)	Lithotomy (*n* = 2), prone jackknife (*n* = 14)	Yes
Marks 2021 [9]	2	Case report	DV single-port	Endoscopically unresectable neoplastic polyps (serrated adenoma, tubulovillous adenoma)	No	None	Avg: 180.5 min (207, 154 min)	Avg: 15 mL (0, 30 mL)	Prone (*n* = 1), modified lithotomy (*n* = 1)	Yes
*Paull 2020 [8]	21	Case series	DV Si (*n* = 10), Flex robotic system (*n* = 11)	Early rectal neoplasia (T0-T1, N0)	Laparoscopic TAMIS (*n* = 3), transabdominal approach (*n* = 1)	DV Si: fractured mass (*n* = 1), proctotomy (*n* = 1). Flex: case aborted (*n* = 1)	DV Si: avg 167.6 ± 84.2 min (range 101–361) Flex: 110.1 ± 39.9 min (range 55–180)	DV Si: avg 37.5 ± 38.3 mL (range 5–100) Flex: 9.1 ± 13.6 mL (range 5–50)	Prone jackknife or high lithotomy for both DV Si and Flex	Yes
*Arnott 2018 [10]	10	Case series	DV S-type	Early stage rectal cancer (T0–T1, N0)	Laparoscopic TAMIS (*n* = 2), traditional TAE (*n* = 1), robotic abdominal approach (*n* = 1)	Rectal stenosis requiring dilation (*n* = 1), peritoneal entry (*n* = 1)	167 ± 26 min (101–361 min)	37.5 ± 11.6 mL (range 5–100 mL)	Lithotomy or prone jackknife	Yes
Ngu 2018 [18]	6	Prospective cohort	DV Xi	Locally advanced rectal carcinoma (cT3/4 ± *N*, 0 M) after neoadjuvant CRT	No	None	Median 106.5 min (range 69–217 min)	Minimal	Modified lithotomy	Yes
Paull 2018 [17]	1	Case report	Flex robotic system	Submucosal anterior mass, suggestive of GIST	No	None	–	–	–	Yes
Atallah 2014 [19]	1	Case report	DV Si	Poorly differentiated adenocarcinoma (pT1)	No	None	93 min	Negligible	Modified lithotomy with moderate Trendelenburg	Yes
Bardakcioglu 2013 [20]	1	Case report	DV	Recurrent rectal adenoma	No	None	–	–	Lithotomy	Yes
Buchs 2013 [12]	3	Prospective cohort	DV Si	Incomplete excision of T1 adenocarcinoma	No	Peritoneal entry (*n* = 1)	Mean 110 min (range 90–120 min, s.d. 17.3)	Mean 6.7 ± 2.9 mL	Left or right lateral position	Yes

*These studies contained overlapping datasets

*DV* Da Vinci, *EBL* estimated blood loss, *TEO* transanal endoscopic operation, *TAMIS* laparoscopic transanal minimally invasive surgery

For all 12 studies combined, indications for R-TAMIS included a benign adenoma or early rectal carcinoma that was unsuitable for endoscopic removal but eligible for local excision (*n* = 7/12); early-stage rectal cancer (T0-T1) that was not explicitly described as unsuitable for endoscopic removal (*n* = 4); and locally advanced rectal cancer (ranging from T0-T4) that was status post-treatment with chemoradiation therapy with clinical remission (*n* = 1). Final pathological diagnoses included benign adenoma, tubulovillous adenoma with or without high-grade dysplasia, villous adenoma, serrated adenoma, rectal adenocarcinoma, gastrointestinal stromal tumor (GIST), carcinoid tumor, and neuroendocrine tumor.

Four of 12 articles reported needing to convert from R-TAMIS to another technique, most commonly transanal endoscopic operation (TEO, *n* = 6), though TEM (*n* = 2) [6, 7], laparoscopic TAMIS (*n* = 3) [8], laparoscopic low anterior resection (*n* = 1) [9], and robotic transabdominal approaches (*n* = 1) [10] were also noted.

Upon removal of the mass, 7 of 12 studies reported negative surgical margins on histology, and only 1 study reported positive margins in 4/26 patients; however, 4 studies did not explicitly comment on surgical margins. Overall, 11 of 12 studies included final surgical pathology (Table 2), most of which was consistent with the pre-operative indication. One study [6] reported a change between pre-operative and post-operative pathology, namely that the pre-operative diagnosis of benign adenoma (*n* = 22/26) and early rectal adenocarcinoma (*n* = 4/26) turned out to be benign adenoma (*n* = 16/26) and rectal adenocarcinoma (*n* = 8/26) with two operations without histology-proven clear resection margins but patients had absence of residual disease.

**Table 2:** Final pathology details from literature review

Author year	*n*	Study design	Robot	Indications	Final pathology	Surgical margins
Morino 2022 [6]	26	Retrospective cohort	Flex robotic system	Benign adenoma (22/26), early rectal adenocarcinoma (4/26) unsuitable for endoscopic removal	Benign adenoma (16/26), early rectal adenocarcinoma (8/26), no histology-proven clear resection margins (2/26)	Positive (*n* = 4), Negative (*n* = 22)
Marks 2021 [7]	26	Phase II clinical trial	DV single port	Benign polyp (16/26), select rectal cancer (10/26) (T1, < / = T2 after neoadjuvant therapy) eligible for local excision	Adenocarcinoma (10/26) with T0 (*n* = 3), T1 (*n* = 3), T2 (*n* = 2), T3 (*n* = 1), T4 (*n* = 1). 7/10 received neoadjuvant chemotherapy; adenoma (14/26), GIST (1/26), carcinoid tumor (1/26)	Negative (*n* = 26)
Studniarek 2021 [13]	1	Case report	DV single port	Rectal neuroendocrine tumor	Rectal neuroendocrine tumor, grade 1 pT1aNxMx	–
Lo 2022 [11]	16	Case series	DV Xi	Endoscopically unresectable tubulovillous adenoma, rectal adenocarcinoma	Negative for tumor (4/16), tubulovillous adenoma (4/16), tubulovillous adenoma with high-grade dysplasia (4/16), invasive rectal adenocarcinoma (4/16)	Negative (*n* = 16)
Marks 2021 [9]	2	Case report	DV single-port	Endoscopically unresectable neoplastic polyps (serrated adenoma, tubulovillous adenoma)	Serrated adenoma (1/2) and tubulovillous adenoma (1/2)	Negative (*n* = 2)
*Paull 2020 [8]	21	Case series	DV Si (*n* = 10), Flex robotic system (*n* = 11)	Early rectal neoplasia (T0-T1, N0)	Neuroendocrine tumor (1/21), tubular adenoma (7/21), villous adenoma (1/21), tubulovillous polyp with adenomatous dysplasia (7/21), GIST (1/21), adenocarcinoma (4/21)	Negative (*n* = 20)
*Arnott 2018 [10]	10	Case series	DV S-type	Early stage rectal cancer (T0-T1, N0)	Neuroendocrine cells (1/10), tubular adenoma (3/10), tubulovillous/villous polyps with adenomatous dysplasia (4/10), adenocarcinoma (2/10)	Negative (*n* = 10)
Paull 2018 [17]	1	Case report	Flex robotic system	Submucosal anterior mass, suggestive of GIST	Low-grade GIST (1/1)	–
Ngu 2018 [18]	6	Prospective cohort	DV Xi	Locally advanced rectal carcinoma (cT3/4 ± N, 0 M) after neoadjuvant CRT	–	–
Atallah 2014 [19]	1	Case report	DV Si	Poorly differentiated adenocarcinoma (pT1)	Sessile serrated adenoma (1/1)	–
Buchs 2013 [12]	3	Prospective cohort	DV Si	Incomplete excision of T1 adenocarcinoma	T1 adenocarcinoma (3/3)	Negative (*n* = 3)
Bardakcioglu 2013 [20]	1	Case report	DV	Recurrent rectal adenoma	Villous adenoma without dysplasia (1/1)	Negative (*n* = 1)

*DV* Da Vinci, *GIST* gastrointestinal stromal tumor, *CRT* chemotherapy

Reported operating room time ranged from a minimum of 45 min to a maximum of 552 min, though median and mean times were around 100–200 min. Of the nine articles that commented on estimated blood loss, three listed EBL as minimal or negligible and the remaining six listed average values between 20 and 40 mL, with absolute ranges from a minimum of 0 mL to a maximum of 100 mL.

Patient positioning varied depending on the location of the lesion. Most studies reported supine, prone, modified lithotomy, or prone jackknife positioning. The supine position was preferred for posterior lesions, and the prone position for anterior lesions. Two studies opted for a left or right lateral decubitus position. 11 of 12 studies, regardless of whether the resection was submucosal or full-thickness, reported suturing the defect closed, most commonly with a 2-0 or 3-0 V-Loc absorbable suture; the remaining study did not explicitly comment on closing the defect.

All but one study detailed their post-operative complications. Pooled together, there were complications in 22/114 patients (19.3%). The listed complications included peritoneal entry (*n* = 7), pelvic or rectal abscess (*n* = 3), local recurrence (*n* = 2), bleeding requiring transfusions (*n* = 2), wound dehiscence (*n* = 1), incontinence (*n* = 1), fractured mass (*n* = 1), proctotomy (*n* = 1), case aborted (*n* = 1), and rectal stenosis requiring dilation (*n* = 1).

Most studies did not specifically comment on post-operative functional outcomes for patients. However, one study noted that no colostomies were needed [9]. Another identified one patient who developed post-operative incontinence at 2 weeks, but this resolved spontaneously after 1 month [11]. A third study described a patient who developed rectal stenosis requiring dilation within 2 months postoperatively after removal of a fragmented mass; this patient did not have evidence of disease recurrence at 6 months [8]. A fourth study included a patient whose procedure had a complication of pneumoperitoneum that was further investigated with exploratory laparoscopy; the patient did not have any post-operative complications and did not develop anal sphincter dysfunction at 2-month follow-up [12].

## Discussion

The included studies described using various robotic platforms, including the Da Vinci Si, Da Vinci Xi, Da Vinci single port, and the flex robotic system. The Da Vinci single port (SP) is a relatively new robotic platform with a single 25-mm cannula that can include a surgeon-controlled 3D camera and three double-jointed articulated arms. The SP has 360-degree rotation of the robotic boom and instruments, thereby allowing access to all quadrants of the rectum without having to reposition the patient or the robot [7]. The SP also avoids external arm collision while working transanally [7, 13]. Having three arms available is advantageous since the third arm can be used for tissue retraction and applying suture tension during wound closure, and if it not needed, can be retracted into the cannula. A holographic navigation system present on the console screen allows the surgeon to monitor the position of up to three instruments. The primary limitations of the SP system are limited instrumentation (namely lack of stapler, suction, and vessel sealer) and a necessary 10-cm distance from the target in order to allow the SP robotic arms to deploy and be fully functional, which becomes challenging when operating on distal lesions [9]. The flex robotic system, which is a relatively new robotic platform featuring a flexible robotic endoscope with two working channels that can accommodate bending instruments (e.g., needle holders, grasping forceps, monopolar tipped or laser holder coagulation instruments), allows for comfortable maneuverability within a long and narrow structure like the colon. The system’s HD-3D visualization also enables a choice between performing an endoscopic submucosal dissection or a full-thickness excision. However, despite the dexterity of the tools, the authors of the study needed to convert to a standard transanal endoscopic operation about a quarter of the time, which the authors attribute to a learning phase and the need for further instrument improvement. The in-progress changes focus on augmenting the dexterity of the endoscope and flexible instruments as well as increasing the availability of different tools, such as a fenestrated grasper, self-adjusting needle holder, and a better endoscope-to-rectoscope seal to prevent air leaks [6].

Variables like patient positioning depended on location of the lesion. One study noted patients with posterior lesions were placed in a supine position while those with anterior lesions were placed in a prone position [6]. One study involving the Da Vinci Xi system at a single community center found patient positioning to be an advantage of a robotic approach over laparoscopic one [11]. While laparoscopic TAMIS becomes more challenging if the lesion is not located in the dependent position, the maneuverability of the robot arms allows access to the lesion regardless of which quadrant it is in. In addition, the primary surgeon preferred placing patients in prone jackknife since it allowed for a wider range of movement for the robotic arms [11]. In contrast, in another study using the da Vinci Xi, the authors were able to routinely place patients in modified lithotomy and docked the robot from the left side of the operating table. They did not experience any difficulty with achieving adequate working angles due to the intraluminal instrument articulation and dexterity [14].

Nearly all included studies had final pathology results consistent with pre-operative indications since patients had already undergone a biopsy and other workup prior to being offered R-TAMIS. A robotic approach allowed for not only improved visualization and surgeon ergonomics, but also access to larger, more proximal, and more complex lesions including circumferential lesions [11].

Since robotic transanal procedures are still in development, a clear approach that would be considered the gold standard is not yet defined. Therefore, we aim to provide a variety of perspectives on how to approach R-TAMIS via a combined literature review and the expertise of a high-volume academic surgeon.

All R-TAMIS procedures at our institution have been performed with the Da Vinci robotic surgical xi system (Intuitive Surgical, Inc., Sunnyvale, CA). This literature review highlights that other robotic platforms are being used to excise rectal lesions. Others have argued that the da Vinci SP single-port platform is a better modality for transanal surgery as it eliminates arm collisions and expands the limits of elbow articulation [15]. We have found that with proper patient and trocar positioning, the Da Vinci Xi platform works well and the Da Vinci SP platform is currently only FDA approved for Head and Neck Surgery and Urology.

### Pre-operative planning

Patients are typically identified as having a rectal lesion on flexible sigmoidoscopy or colonoscopy. Pre-operative MRI abdomen/pelvis is helpful in determining the distance from the anal verge as well as potential invasion beyond the submucosa [16]. A digital rectal exam and rigid proctoscopy is also helpful in determining the location of the lesion. Moreover, the relationship of the anterior lesions to the middle valve of Houston on MRI is important as it corresponds anteriorly to the peritoneal reflection. If the lesion is above this reflection, a full-thickness resection could result in an intra-peritoneal defect. We recommend that the patient should undergo a mechanical bowel preparation with oral antibiotics (Neomycin, Flagyl) the day prior to surgery.

### Operative setup

**Fig. 1:**
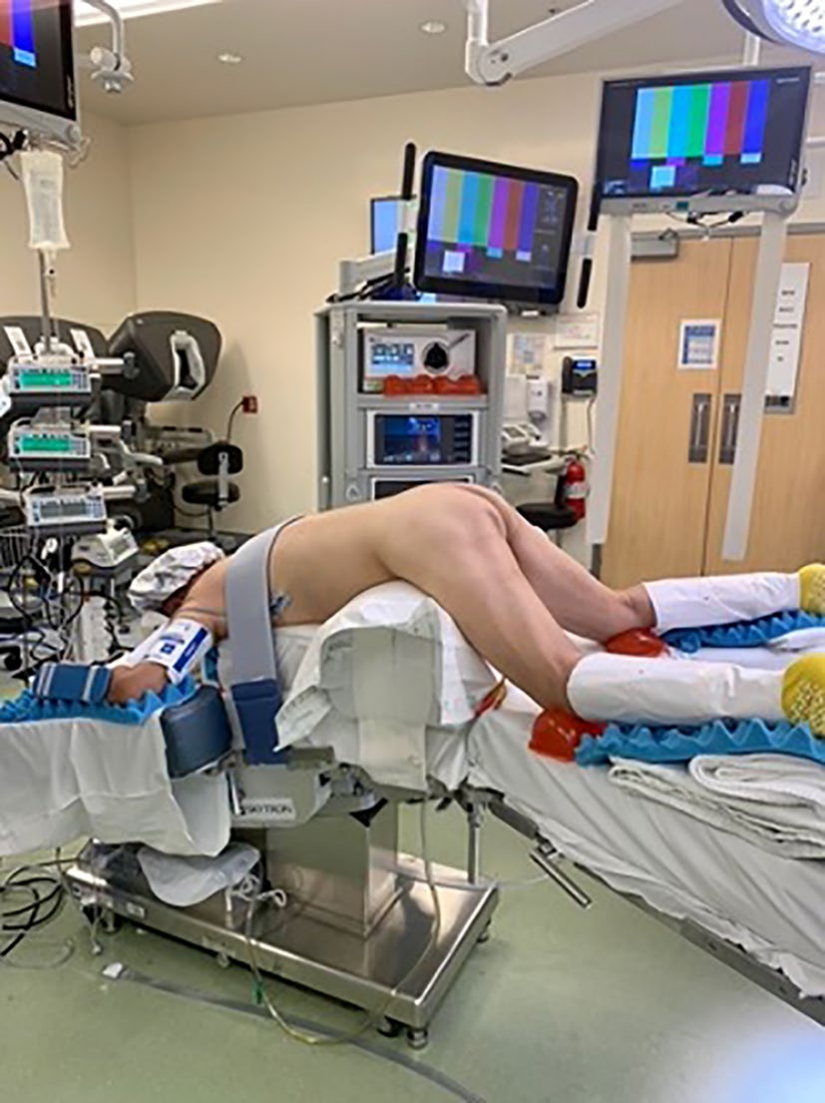
A patient positioned in prone jackknife. This prevents the patient’s legs from colliding with the robotic arms

**Fig. 2:**
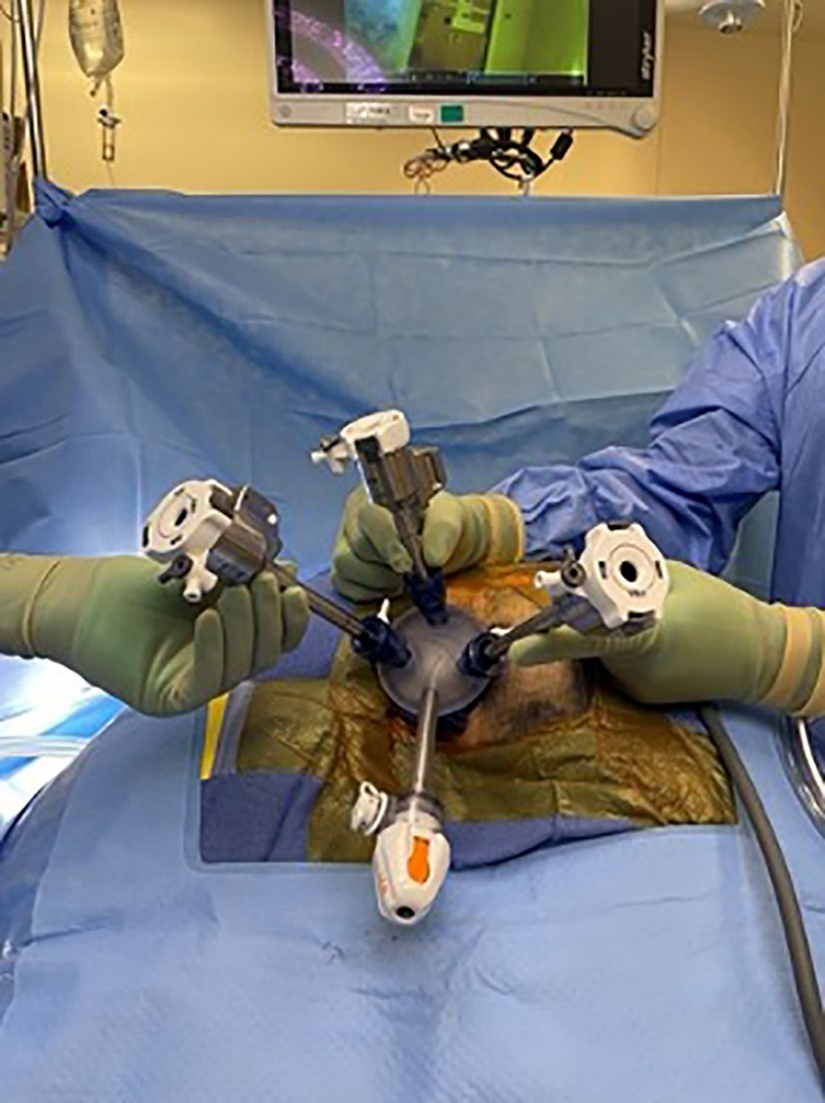
Placement of trocars through the GelPort

**Fig. 3:**
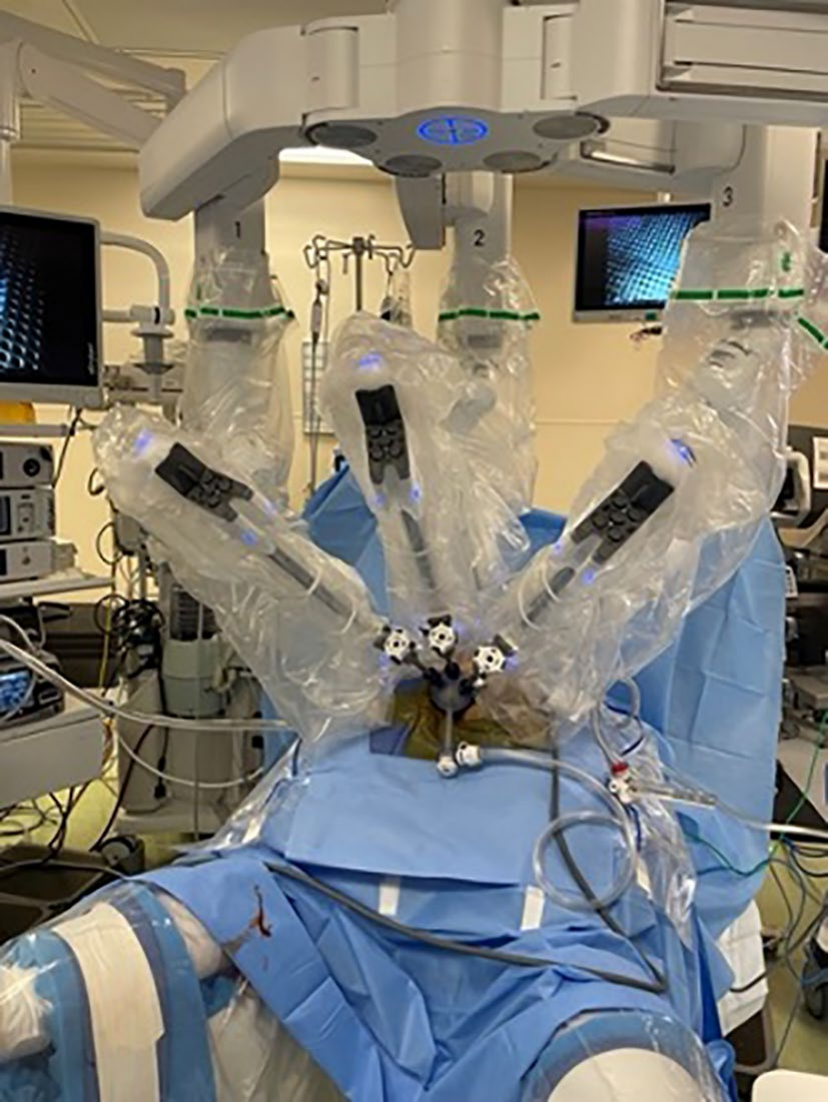
Robot placement at the patient’s side so that direct access to perineum in between the legs is maintained

We recommend general anesthesia to enable full muscle relaxation and rectal insufflation. A urinary catheter is not necessary. A single dose of pre-operative antibiotic is given. The optimal position is often prone jackknife. Positioning the patient in prone jackknife prevents the patient’s legs from colliding with the robotic arms (Fig. 1). The GelPOINT path device (Applied Medical, Rancho Santa Margarita, CA, USA) is inserted transanally and sutured to the skin of the buttocks. The standard 8 mm da Vinci trocars are then placed through the GelPort (Fig. 2). A 5 mm AirSeal (CONMED Corporation, Utica, NY, USA) is also inserted, for insufflation and manual assistance by a bedside assistant. Use of the AirSeal helps maintain stable pneumoinsufflation of the rectum. The robot should be brought in from the patient’s side, so that direct access to the perineum in between the legs is still available for the surgeon or assistant (Fig. 3).

### Surgical technique

**Fig. 4:**
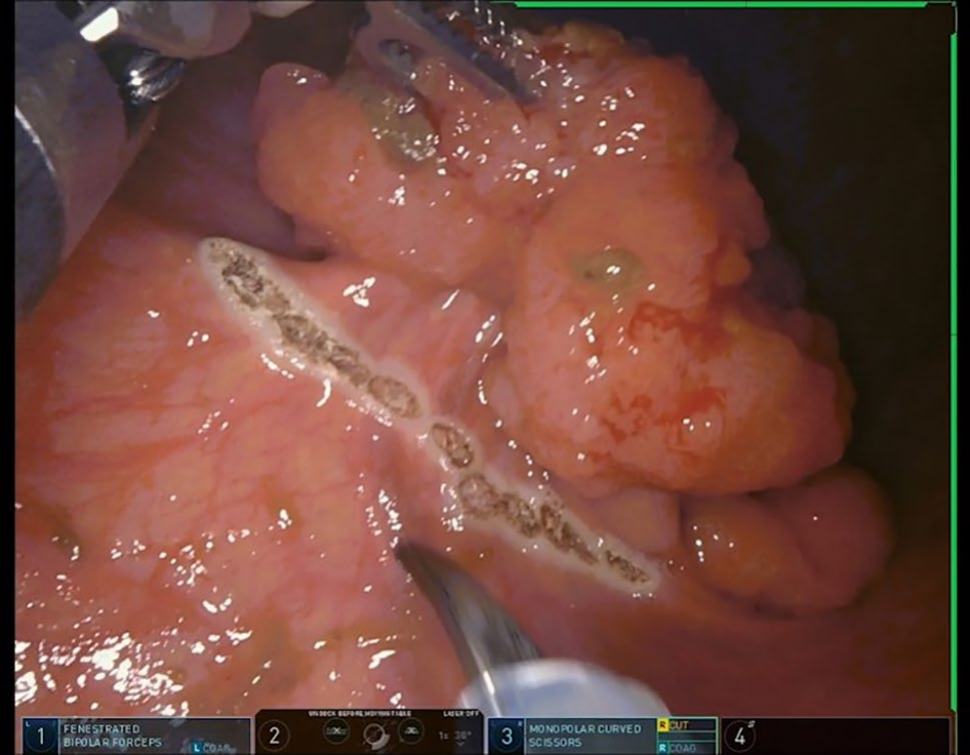
The lesion is circumferentially outlined with a 1 cm margin using electrocautery

**Fig. 5:**
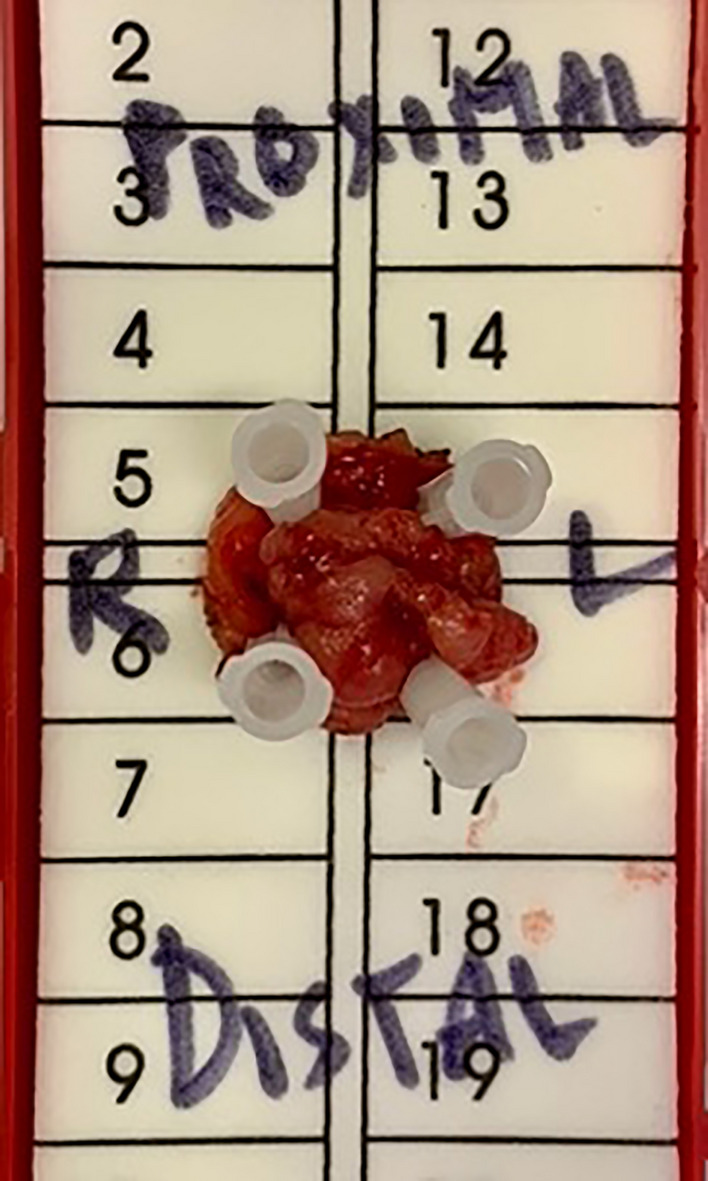
Intact specimen removed and marked with correct orientation for final pathology

Placing a Raytec proximal to the lesion prevents effluent from coming down. The rectal lesion should be positioned inferiorly on the screen if possible to optimize wrist movement. The lesion is circumferentially outlined with a 1 cm margin using electrocautery prior to resection (Fig. 4). A full-thickness resection maintaining the relationship of all the layers of the rectal wall is performed. The defect is then closed primarily. We prefer using a running 6-inch V-Loc^™^ (Medtronic, Minneapolis, MN) suture as it can prevent knot-related complications and reduce suturing time [17, 18]. We found backhand suturing to be the most ergonomic and efficient technique to take full-thickness bites. While bleeding is uncommon, if significant it can be controlled using the Vessel Sealer or Ligasure. The specimen is removed intact and marked with correct orientation for final pathology (Fig. 5). Leaving a drain is not necessary.

### Post-operative care

Robotic TAMIS can typically be done as outpatient, with the patient going home on the day of surgery. All patients are seen in clinic at 2 weeks for follow-up, with further follow-up dependent on the final pathology.

In conclusion, we review the literature pertaining to robotic TAMIS and provide practical tips and tricks for surgeons interested in using the robot for these types of cases.
